# Establishment of an efficient reverse genetic system of Mumps virus S79 from cloned DNA

**DOI:** 10.1007/s12519-019-00286-8

**Published:** 2019-08-28

**Authors:** Duo Zhou, Meng-Ying Zhu, Yi-Long Wang, Xiao-Qiang Hao, Dong-Ming Zhou, Rong-Xian Liu, Chu-Di Zhang, Chu-Fan Qu, Zheng-Yan Zhao

**Affiliations:** 1 Zhejiang University School of Medicine, Hangzhou, China; 2 Children’s Hospital, Zhejiang University School of Medicine, Hangzhou, 310052 China; 3 Department of Neurology, Children’s Hospital, Zhejiang University School of Medicine, Hangzhou, 310052 China

**Keywords:** Mumps virus, Reverse genetics, Plasmid, Safety, Immunogenicity

## Abstract

**Background:**

Mumps is a common type of respiratory infectious disease caused by mumps virus (MuV), and can be effectively prevented by vaccination. In this study, a reverse genetic system of MuV that can facilitate the rational design of safer, more efficient mumps vaccine candidates is established.

**Methods:**

MuV-S79 cDNA clone was assembled into a full-length plasmid by means of the GeneArt™ High-Order Genetic Assembly System, and was rescued via reverse genetic technology. RT-PCR, sequencing, and immunofluorescence assays were used for rMuV-S79 authentication. Viral replication kinetics and in vivo experimental models were used to evaluate the replication, safety, and immunogenicity of rMuV-S79.

**Results:**

A full-length cDNA clone of MuV-S79 in the assembly process was generated by a novel plasmid assemble strategy, and a robust reverse genetic system of MuV-S79 was successfully established. The established rMuV-S79 strain could reach a high virus titer in vitro. The average viral titer of rMuV-S79 in the lung tissues was 2.68 ± 0.14 log_10_PFU/g lung tissue, and rMuV-S79 group did not induce inflammation in the lung tissues in cotton rats. Neutralizing antibody titers induced by rMuV-S79 were high, long-lasting and could provide complete protection against MuV wild strain challenge.

**Conclusion:**

We have established a robust reverse genetic system of MuV-S79 which can facilitate the optimization of mumps vaccines. rMuV-S79 rescued could reach a high virus titer and the safety was proven in vivo. It could also provide complete protection against MuV wild strain challenge.

## Introduction

Mumps is a common type of respiratory infectious disease caused by mumps virus (MuV) [1] characterized by swelling of the parotid gland. MuV can also lead to orchitis, deafness, sterile meningitis, and encephalitis [2]. There is still no effective treatment, specifically for mumps. The effective way to prevent mumps is vaccination. In China, live-attenuated S79MuV vaccine originated from Jeryl Lynn strain has been licensed for vaccination since 1990, and the measles–mumps–rubella virus (MMR) vaccine was introduced into the National Immunization program in 2008 for the control of measles, mumps, and rubella [3, 4]. Although high coverage with MMR, incidence of mumps remained high after the one-dose measles–mumps–rubella (MMR) vaccine in China [4–6]; much more efforts are needed to seek for improved MuV vaccine candidates with restricted replication, sufficient immunogenicity, and reduced reactogenicity.

MuV is an enveloped, non-segmented, negative-sense (NNS) RNA virus in the family Paramyxoviridae, subfamily Paramyxovirinae, genus Rubulavirus.  The MuV genome encoded seven transcription units: the nucleo (N), V/phospho/I (V/P/I), matrix (M), fusion (F), small hydrophobic (SH), hemagglutinin neuraminidase (HN), and large (L) protein genes [7–9]. Similar to other NNS RNA viruses, the ribonucleoprotein (RNP) complex is the minimal machinery for transcription and replication of MuV, which consists of the nucleocapsid (N)-RNA template tightly associated with the RNA-dependent RNA polymerase, the large (L) polymerase protein, and the phosphoprotein (P) [10]. The nucleocapsid (N)-RNA is the template for RNA synthesis, and only encapsidated, but not naked RNA, can be transcribed. RNA synthesis begins with the binding of the RNA-dependent RNA polymerase (RdRp), a complex of the P and L proteins, to the NP protein-gRNA [11–16]. Recently, using reverse genetics to derive attenuated derivatives of wild-type RNA virus strains or live-attenuated RNA virus vaccine strains, creation of safer and highly effective vaccine candidates for MuV is allowed as for other NNS RNA viruses [11, 13–17]. Plasmids that can express viral anti-genome RNA, viral NP, P, and L proteins under the control of the T7 RNA polymerase promoter are essential for the recovery of MuV. When recovery succeeds, cDNA-rescued MuV could be propagated in the same manner with parental virus. And the rescued virus is able to differentiate from parental virus by introducing mutations as gene tag in the virus genome [12, 18]. The reverse genetic system of MuV can facilitate the rational design of safer, more efficient mumps vaccine candidates.

In this study, a novel, more efficient method was used to construct a full-length cDNA clone of MuV-S79 without the need for restriction endonucleases in the assembly process and rMuV-S79 was successfully recovered in BHK cells stably expressing T7 RNA polymerase. Our results showed that rMuV-S79 reached a high virus titer in vitro, and did not induce inflammation changes in lung tissues in cotton rats. Neutralizing antibody titers induced by rMuV-S79 were high, long-lasting and could provide complete protection against MuV wild strain challenge.

## Methods

### Cells and MuV

BHK-SR19-T7 cells [19] (kindly provided by Apath, LLC, Brooklyn, NY) and Vero cells (ATCC) were cultured in DMEM (Life Technologies, USA) culture medium added with 10% fetal bovine serum (FBS, Life Technologies, USA). The S79 strain of MuV (kindly provided by Professor Yiyu Lu, Zhejiang CDC, China) was passaged in Vero cells, and wide-type MuV was isolated from patients in Children’s Hospital, Zhejiang University School of Medicine after obtaining the written informed consents from the parents.

### Construction of MuV-S79 genomic plasmids

Viral RNA of MuV-S79 extracted with a RNeasy mini-kit (Qiagen, Germany) was reverse-transcribed into cDNA with Super Script® III reverse transcriptase (Life Technologies, USA) according to the manufacturer’s instructions. MuV genome was amplified with five pairs of MuV-specific primers (Table 1) dividing the genome into five overlapping fragments was designed using Q5® High-Fidelity 2X Master Mix (NEB, USA) and the DNA fragment was inserted into the pEASY-Blunt vector plasmid (Transgen, CHINA) following the manufacturer’s instructions. Five resultant plasmids covering the full-length MuV-S79 genome (pEASY-MuV-NP-P, pEASY-MuV-M-F, pEASY-MuV-SH-HN, pEASY-MuV-L1, and pEASY-MuV-L2) were send for sequencing. The sequence of MuV-S79 in our lab was submitted to Genbank with accession FJ416067.

**Table 1:** Primers sequences used for virus genome PCR

Primers	Sequence (5′–3′)
MuV-NP-5endF	ACCAAGGGGAAAATGAAGATG
MuV-NP-R	CTTGAGTCTGGTGCTTCTGGTG
MuV-M-F-F	CACCGAGGATGCTCTGAACGAC
MuV-M-F-R	TAAGGAGGGTTGGATTGCCG
MuV-SH-HN-F	CTAGGGTCGTAACGTCTC
MuV-SH-HN-R	TAAGAAATGAGACACGCC
MuV-L1-F	GAGTTGTAGTGAATGTAGTAGG
MuV-L1-R	GTATTCTATTACCGTATTCAGC
MuV-L2-F	AGACCACTGTCAGCAAAG
MuV-L2-R	ACCAAGGGGAGAAAGTAG

### Construction of the full-length cDNA clone of MuV-S79 and the supporting plasmids for MuV-S79 NP, P, and L proteins

**Fig. 1:**
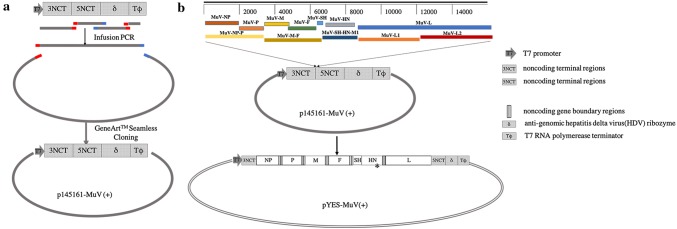
Schematic representation of the pYES-MV (+). Four fragments, T7 promoter, 3′ and 5′ non-coding termini (NCT), anti-genomic HDV ribozyme and T7 terminator, were introduced into pYES-2 through seamless assembly after several rounds of fusion PCR, resulting in p145161-MuV (+) (a). The full-length MuV genome consisted of five overlapping fragments was assembled into p145161-MuV (+), creating pYES-MuV (+) (**b**). A silent change (C to U) in HN gene that distinguishes rescued recombinant virus (marked by “*”) from the parental virus strain in our laboratory

DNA fragment contained with MuV 3ʹ and 5ʹ non-coding termini (NCT) (3′–145 nt and 5′–169 nt, respectively), anti-genomic hepatitis delta virus (HDV) ribozyme sequence, the T7 promoter, and the T7 terminator were amplified with in-fusion PCR and then was cloned into the pYES-2 plasmid to generate with plasmid p145161-MuV (+) with the GeneArt™ Seamless Cloning and Assembly Kit (Invitrogen, USA; Fig. 1a). The sequences of primer and approaches utilized in the PCR assays are available in needed upon request. Two fragments containing the full length of p145161-MuV (+) was amplified with specific primers (F:5′-TTCTGCCGCCTGCTTCAAACCG-3′, R:5′- TTTCCAGGTAGTGTCAAAATG-3′; F:5′-ATCGAATAAAGACTCTTCTG-3′,R:5′-CAGAATGGGCAGACATTACGAATGC-3′). Five fragments containing the MuV-S79 genomic were amplified with specific primers from pEASY-MuV-NP-P, pEASY-MuV-M-F, pEASY-MuV-SH-HN, pEASY-MuV-L1, and pEASY-MuV-L2. Plasmid pYES-MuV (+), a full-length cDNA clone of MuV-S79, was successfully generated by assembling seven overlapping fragments with GeneArt™ High-Order Genetic Assembly System (Fig. 1b).

The strategy to construct the supporting plasmids of pT7-MuV-S79-NP, pT7- MuV-S79-P, and pT7-MuV-S79-L using “seamless” cloning had showed in the previous study [12, 20, 21]. The sequence of primers used in the PCR assays is available on request.

### Generation of infectious rMuV-S79 from transfected cells

BHK-SR-19-T7 cells in 6-well plates with cell density of 90% confluence were transfected with 5 µg of pYES-MuV (+), 1.5 µg of pT7-MuV-S79-NP, 0.25 µg pT7-MuV-S79-P, and 2.5 µg pT7-MuV-S79-L [22–24]. At 6 hours post-infection (hpi), cell transfection mixture was discarded and cell was cultured with Opti-MEM. At 72 hours post-transfection, confluent cell monolayers were directly transferred onto Vero cell monolayers (P0) at 75% confluence and incubated at 37 °C for 2–4 days. Extensive CPE (MuV-induced syncytia) could be visualized under microscope if successes. Supernatants (P1) from Vero cells were further passaged on confluent Vero cell monolayers and incubated for 3–5 days, and the virus were verified with immunofluorescence assay using mouse anti-Mumps nucleoprotein (ab106292, abcam).

### Confirmation of sequences by RT-PCR

All the plasmids, viral stocks were sequenced. A 0.71 kb DNA region of the HN protein gene containing the gene tag was amplified by a one-step RT-PCR kit (Qiagen, Germany) using primers MuV-HN-7634-Forward (5′-GAGAATTTTTCCGGCCCGTT-3′) and MuV-L-8342-Reverse (5′-CAAGTGATGGTCAATCTGGC-3′). The amplified products were analyzed on 1% agarose gel and sequenced.

### Viral replication kinetics in Vero cells

Confluent Vero cells were grown in 6-well plates and then were inoculated with MuV at a multiplicity of infection (MOI) of 0.01, 0.1, and 1. After virus absorption for 1 h, the cells were washed three times with PBS. Infected cells were incubated at 37 °C incubator and then the cells were subjected to three freeze–thaw cycles, and the supernatant of MuV was harvested by centrifugation at 3000×*g* in an Eppendorf 5804R centrifuge for 10 min. Virus titers were detected in Vero cells using plaque assay according to our previous study [20].

### Replication of rMuV-S79 in cotton rats

Ten 4–6-week-old female specific-pathogen-free (SPF) cotton rats (kindly provided by Professor Enmei Liu from Children's hospital of Chongqing medical university) were randomly divided into two groups (each group with five cotton rats). Cotton rats of each group were inoculated with rMuV-S79, and Opti-MEM respectively. Each cotton rat was inoculated intranasally with 1 × 10^6^ PFU of virus in a volume of 100 μl. At 4 dpi, cotton rats were sacrificed and lungs were collected for virus titration and did pulmonary histopathology.

### Immunogenicity of rMuV-S79 in cotton rats

Cotton rats (kindly provided by Professor Enmei Liu from Children's Hospital of Chongqing Medical University) between 4 and 6 weeks of age were divided into two groups, infected with rMuV-S79 (five cotton rats) and Opti-MEM (five cotton rats), respectively. The rats were anesthetized and vaccinated with viruses intranasally. Blood samples were obtained by retro-orbital puncture after anesthetized at week 3, week 4, week 5, week 7, and week 9 after vaccination. Serum neutralization of virus was detected using an endpoint dilution plaque reduction assay. At 4 week post-immunization, the cotton rats were challenged with 1.0 × 107 PFU of wild-type MuV and the presence of any clinical symptoms were evaluated twice daily. At 4 day post-challenge, all cotton rats were sacrificed and their lungs were collected for virus titration.

### Statistical analysis

Statistical analysis was analyzed by one-way multiple comparisons utilizing Prism, version 8.0, statistical analysis software. *P* value of < 0.05 was considered statistically significant.

## Results

### Recovery of rMuV-S79 from a full-length cDNA clone

**Fig. 2:**
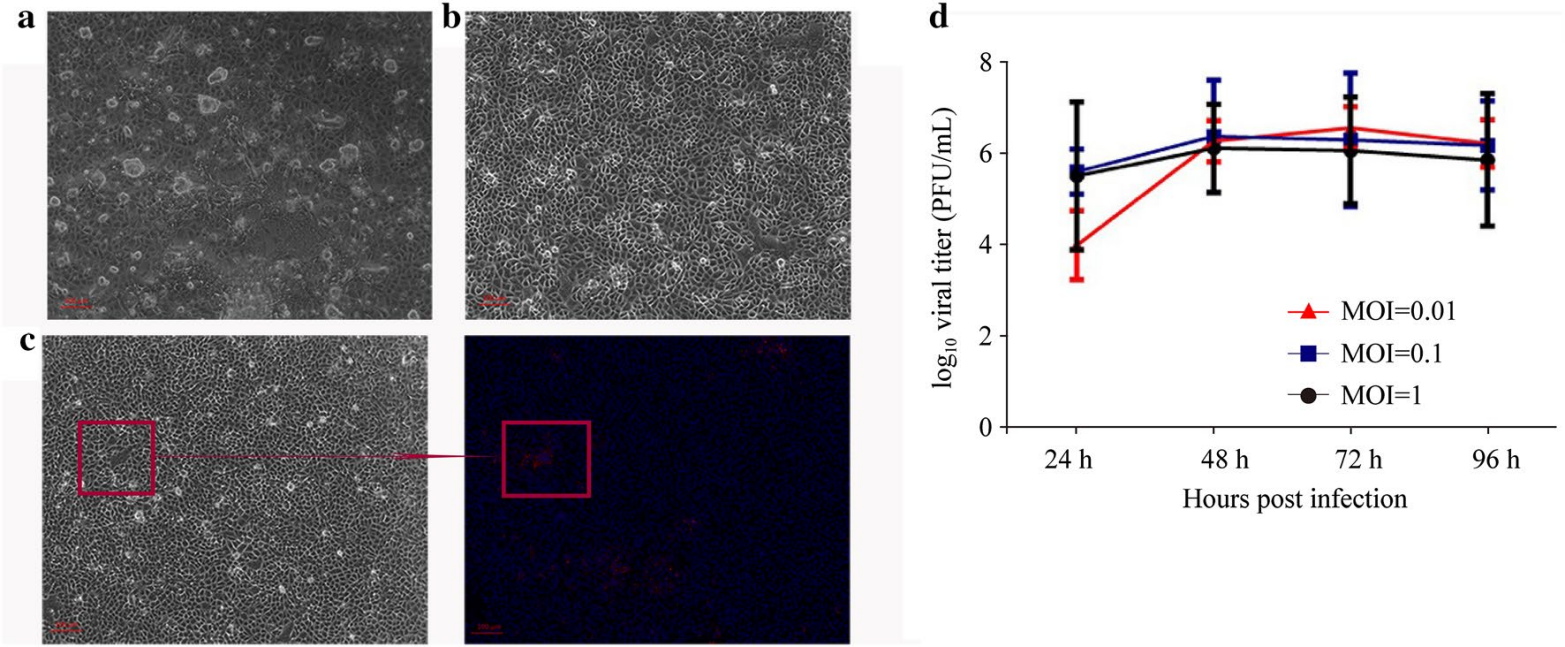
pYES-MuV (+) plasmid and helper plasmids pT7-S79-NP, pT7-S79-P, and pT7-S79-L were transfected into BHK-SR19-T7 cells. Transfected BHK-T7 cells were co-cultured with Vero cells on day 3. CPE was observed after 48 h of coculture (**a**). The rescued virus supernatant (P1) were further passaged onto Vero cells, and incubated for 24 h (**b**). The successful recovery of rMuV-S79 was further confirmed by detection of NP protein expression in Vero cells infected with the rescued rMuV-S79 by immunofluorescence assay (**c**). Vero cells on 6-well cell culture cluster were infected with viruses at MOI of 1, 0.1, and 0.01, and collected at different time points (24 h, 48 h, 72 h and 96 h). After three freeze–thaw cycles, virus titers were determined by plaque assay in Vero cells. Virus growth curves are shown **d**

pYES-MuV (+), a MuV-S79 cDNA clone, was successfully established with the GeneArt™ High-Order Genetic Assembly System [25]. Figure 1 illustrates a schematic representation of the full-length plasmid pYES-MuV (+) which under the control of a T7 RNA polymerase promoter, hepatitis delta virus (HDV) ribozyme sequence, and T7 terminators [25]. BHK-SR-19-T7 cells stably expressing T7 RNA polymerase were transfected with pYES-MuV (+), pT7-S79-NP, pT7-S79-P, and pT7-S79-L to rescue infectious MuV from cDNA. On day 3 post-transfection, the cell monolayers were harvested and directly transferred onto Vero cell monolayers at 70–80% confluence. MuV-induced syncytia was observed 2–3 days afterwards (Fig. 2a).

### Identification of rMuV-S79

To confirm the rescued rMuV-S79, we detected the expression of NP protein on Vero cells which were infected with the rescued rMuV-S79 in 24-well plates by immunofluorescence assay (Fig. 2c). Gene tag represented silent changes at nucleotide (nt) position 8134 (C to T) which in HN gene introduced in pYES-MuV (+). To verify that the rMuV-S79 was derived from cDNA but not cross-contamination from the MuV-S79 parental strain grown in our laboratory, the regions spanning the nucleotide tag were amplified by RT-PCR and sent for sequencing. Sequence results of RT-PCR products showed that the recovered virus was from pYES-MuV (+).

### Viral replication kinetics of rMuV-S79 in Vero cell

Replication kinetics of rMuV-S79 in Vero cells was confirmed. As demonstrated in Fig. 2d, rMuV-S79 infected Vero cells at MOI of 0.01, 0.1, and 1. At MOI of 0.1 and 1, the virus titers of rMuV-S79 reached the highest level 48 h post-infection (hpi) in Vero cells, and the virus titers reached the highest level at 72 hpi at MOI of 0.01.

### Safety of rMuV-S79 in cotton rats

To evaluate the virus replication of rMuV-S79 in vivo, specific pathogen-free cotton rats were divided into two groups vaccinated with rMuV-S79 and Opti-MEM, respectively. No clinical symptoms of respiratory tract infection appeared in cotton rats inoculated with any of the rMuVs. Four days after inoculation, cotton rats were sacrificed to extract left lung tissues for detecting the viral titer (Table 2). The average viral titer of rMuV-S79 in lung tissues was 2.68 ± 0.14 log_10_PFU/g (Table 2).

**Table 2:** Replication of rMuV-S79 in the cotton rats

Groups	Viral replication in lung	
	% infected animals	Viral titer (log_10_ PFU/g)
rMuV-S79	100	2.68 ± 0.14
Opti-MEM	0	Not detected

Five cotton rats were inoculated with rMuV-S79 via intranasal route. Five cotton rats in control group were received the same volume of Opti-MEM without virus. Two groups of cotton rats were terminated at 4th day post-inoculation and viral titers in lung tissues were determined by plaque assay

**Fig. 3:**
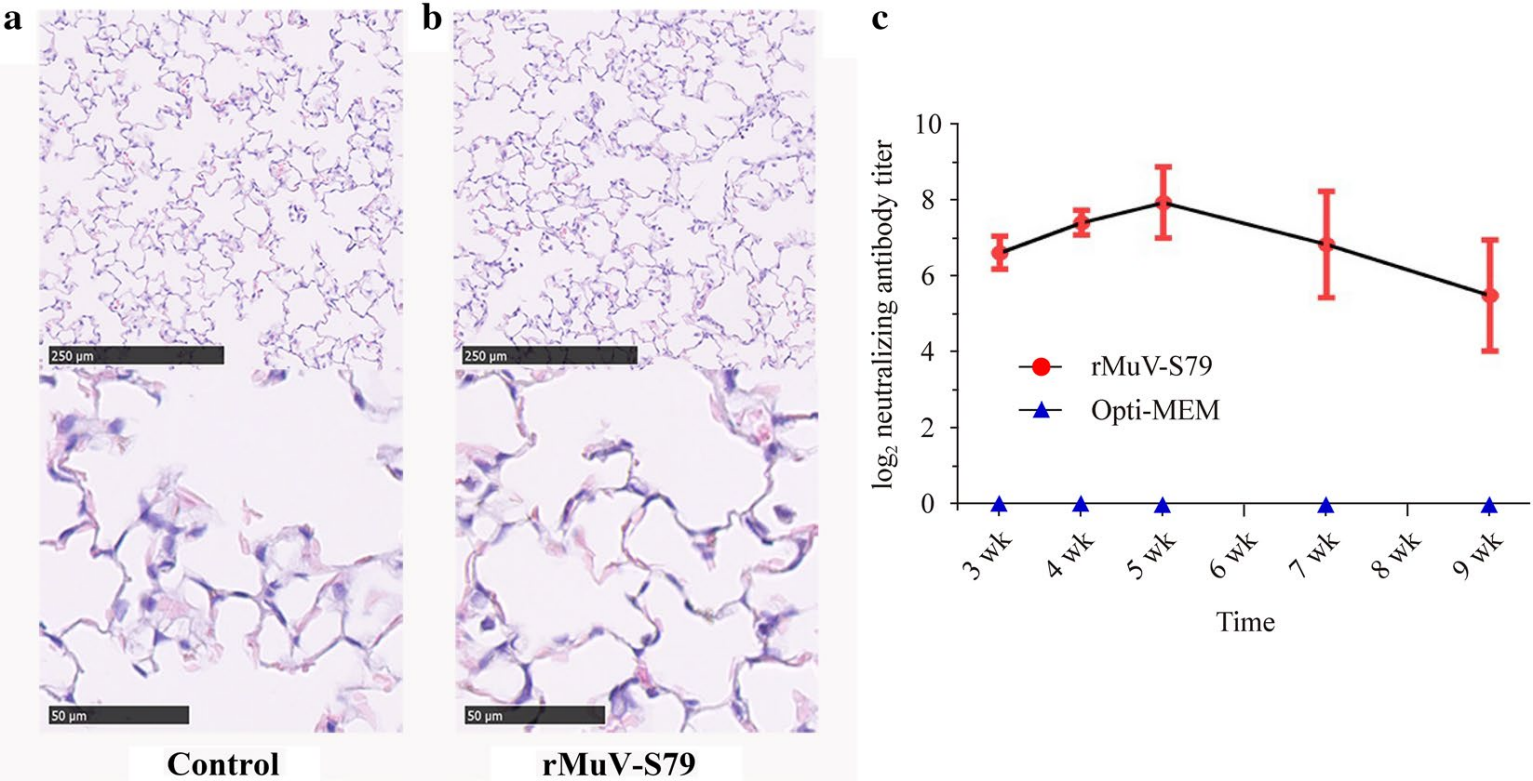
Cotton rats were grouped infected with 10^6^ PFU of rMuV-S79. Five cotton rats were inoculated Opti-MEM as control group. Histological examination was conducted by HE staining of lung tissues from vaccination group (**a**) and control group (**b**) at 4th day post-inoculation. Sera were obtained at 3–5, 7, and 9 weeks after immunization to detect neutralization anti-MuV antibodies. The antibodies titers at each time point quantified by 50% plaque reduction assay are calculated and shown as mean NT titers (**c**)

Histological examinations of lung tissues obtained from cotton rats 4 days after vaccination were performed. Histological sections of lung tissues after hematoxylin and eosin staining (HE staining) are shown in Fig. 3a. There was no significant morphological difference between the rMuV-S79 and Opti-MEM groups. Lung tissues of the rMuV-S79 group did not show signs of inflammation in comparison with the control group.

These results indicated that the rMuV-S79 recovered was safe in vivo.

### Vaccine immunogenicity of rMuV-S79

Cotton rats (4–6 weeks old) (five animals per group) were inoculated with rMuV-S79 via the intranasal route, and the control group received the same volume of Opti-MEM (Table 3). The serum samples were collected at weeks 3, 5, 7, and 9 after immunization for analyzing the immunogenicity of rMuV-S79. To examine the neutralizing antibody titers against MuV, the neutralization test (NTs) was conducted with the 50% plaque reduction assay. Our results demonstrated that neutralizing antibody titers induced by rMuV-S79 peaked at week 5 with a titer of 7.92 log2 neutralizing antibody titer and lasted a long time. The neutralizing antibodies could provide complete protection against MuV wild strain challenge (Fig. 3b). At week 9 post-vaccination, cotton rats were inoculated with 1.0 × 10^7^ PFU of wild-type MuV and all cotton rats were sacrificed at day 4 post-challenge. No infectious virus was detected in the lung tissue in the rMuV-S79 vaccinated cotton rats; however, an average titer of 3.35 ± 0.26 log10 PFU/g was detected in lung tissue from uninoculated but challenged controls (Table 4).

**Table 3:** Cotton rats were grouped to test neutralizing anti-MuV antibodies in serum

Groups (5 per group)	Route	Vaccine	Dose (PFU/ml)
rMuV-S79	I.N.	rMuV-S79	1 × 10^6^
Control	I.N.	Opti-MEM	Opti-MEM only

*I.N.* intranasal

**Table 4:** Immunogenicity of rMuV-S79 in cotton rats

Groups	Viral replication in lung	
	% infected animals	Viral titer (log_10_ PFU/g)
rMuV-S79	0	Not detected^A^
Opti-MEM	100	3.35 ± 0.26^B^

Five cotton rats in vaccination group were intranasally inoculated with 1.0 × 10^6^ PFU and the blanket control group was received Opti-MEM of the same volume. Both groups of cotton rats were challenged with 1 × 10^7^ PFU of wild-type MuV after 9 week post-infection and sacrificed at day 4 post-challenge to collect lung tissues for virus titration assay and RT-PCR

Values within a column followed by different capital letters (A and B) are significantly different

## Discussion

Symptoms caused by MuV are normally mild, short-living and disappear without sequelae. However, serious complications like sensorineural or encephalitis deafness may occur. Mumps still occur in people who previously had one or two doses of MMR vaccine, driving us to develop a safer and more efficacious vaccine. Efficient reverse genetic system of negative-strand RNA viruses has provided a new approach for the study of virus growth, and has been used in the prevention and treatment of disease.

In the early studies, the common strategy applied to construct full-length cDNA of MuV, measles virus was stepwise cloning using restriction enzyme. In this research, full-length cDNA clone of MuV-S79 was constructed using a novel plasmid assemble strategy which restriction and ligation were not needed for generating plasmid in the assembly process by using a GeneArt™ High-Order Genetic Assembly System. Based on this novel assembly system, full-length genomic clone of measles virus has been generated in our previous research [20].

In the past, common strategies to rescue MuV using reverse genetic systems were based on the vaccinia helper viruses which could express T7 RNA polymerase [12]. However, it is difficult to separate the rescued MuV from the helper viruses. Stably-transfected BHK cells expressing T7 RNA polymerase have been used to rescue bovine respiratory syncytial virus, hMPV and measles virus [19, 26]. In this study, BHK-SR-19-T7 cells were co-transfected with a plasmid expressing cDNA genome of MuV and supporting plasmids expressing the MuV NP, P, and L proteins. The success of this efficient recovery process lies in vaccinia virus-freeness. The rescued viruses generated from this process were passaged for fewer times than biologically derived virus with reducing possibility of adventitious contamination.

Replication kinetics, safety, and immunogenicity of rMuV-S79 were confirmed both in vitro and in vivo. We found that rMuV-S79 were well replicated in Vero cell and did not induce inflammation changes in lung tissues in cotton rats. Our results showed that vaccination with rMuV-S79 could provide complete protection against MuV wild strain challenge.

Reverse genetics systems are helpful for virus research and can provide vaccine candidates. With a robust reverse genetic system, virus can be engineered with mutations, insertions, and deletions. In this study, an efficient reverse genetic system of MuV-S79 was generated, providing the potential to engineer the virus genome to express foreign genes, as described for measles viruses [27, 28]. Novel recombinant MuV vaccine candidates expressing foreign gene at different loci of the MuV-S79 genome could be constructed in future study based on this efficient reverse genetic system.
